# Literary Notes

**Published:** 1897-05

**Authors:** 


### Literary Notes.

Military Cycling in the Rocky Mountains, by Lieut. James A. Moss, commander of the Twenty-fifth U. S. Infantry Bicycle Corps, is the title of No. 62 of Spalding's Athletic Library. It contains an interesting account of the trips of the first bicycle corps organised in the army and, besides a handsome portrait of Gen. Miles, is illustrated with views taken in Yellowstone Park and along the line of march. The book will be sent postpaid to any address in the United States or Canada on receipt of 10 cents by the American Sports Publishing Co., 241 Broadway, New York.

---

The Sheppard Asylum, a hospital for mental diseases, located near Towson, Md., has lately issued its fifth annual report. It is a handsome brochure, well printed and beautifully illustrated with views of the buildings and grounds. The late Enoch Pratt left to the institution a bequest making the trustees of the asylum his residuary legatee, providing his own name shall be hereafter coupled with that of Mr. Sheppard in the title of the institution. Dr. E. N. Brush, a former Buffalonian, is the medical superintendent, and his valuable services are well recorded in this report.

---

The *Fort Wayne Medical Journal-Magazine* represents a consolidation of the interests of the *Journal of the Medical Sciences*, established sixteen years ago, and the *Fort Wayne Medical Magazine*, established four years ago. The editorial staff of the consolidated periodical includes all of the editors of both journals, the editor of the *Fort Wayne Medical Magazine* having been chosen editor-in-chief. The first issues of this new enterprise present an excellent appearance and we have no doubt the change will prove beneficial to all concerned.

---

THE *Columbus Medical Journal*, our most excellent Ohio semi-monthly contemporary, has removed from 150 E. Broad street to 68 Buttles avenue, Columbus, O.

---

THE *Medical Record of Mississippi*, edited and published at Biloxi by Dr. H. H. Haralson, made its first appearance in April, 1897. Its first number is a creditable one and the journal ought to prove a benefit to a large region heretofore unrepresented in the journalistic field.

---

*Treatment* is the name of a new journal of practical medicine and surgery, published every second and fourth Thursday, at 11 Adam street Strand, London, by Rebman Publishing Company, at the annual subscription of 12 shillings ($3.00), post free. It is admirably edited and printed and contains fresh material relating to the treatment of disease in all the various branches of medical science. Judging from the numbers already issued, *Treatment* is destined to become one of the best exponents extant of therapeutics.

---

THE *Intercollegiate Medical Journal*, published every second month and devoted to the study of the medical sciences, is the official organ of the Nu Sigma Nu fraternity. It is edited by Drs. F. Gurney Stubbs and Will Walter, of Chicago, with the coöperation of an associate staff of twelve assistant editors for the several chapters represented. It is handsomely printed on antique paper and its initial number, March, 1897, indicates an interesting addition to the high-class periodical medical literature of the United States.

---

THE *Boletim da Sociedade de Medicina e Cirurgia de Sao Paulo* (Brazil) pays the BUFFALO MEDICAL JOURNAL an appreciated compliment in one of its recent numbers as follows :

> The BUFFALO MEDICAL JOURNAL, *Bulletin of the Johns Hopkins Hospital* and *Bulletin of the American Academy of Medicine* reach us with the most rigorous punctuality. Their greatly valued and important scientific articles render these highly esteemed journals a credit and likewise an ornament to our science of medicine.

THE Jackson Sanatorium, at Dansville, has lately sent out a year book for 1897, which is a beautiful octavo pamphlet of fifty pages, from the art printing works of Matthews-Northrup Co., Buffalo. It is printed on heavy and highly finished book paper and contains numerous illustrations from photographs, printed in color, of scenes in and about the sanatorium. The year book will be sent on application to Dr. James Arthur Jackson, secretary, Dansville, N. Y.

---

DR. WILLIAM H. HUMISTON, of Cleveland, has recently posted to his professional friends a handsomely printed and illustrated brochure, entitled A Year's Work in Operative Gynecology. It is a creditable exhibition of skilful surgical treatment in a private hospital for women by a progressive gynecological surgeon.

---

RESECTION of arteries and veins injured in continuity—end-to-end suture. Experimental and clinical research—is the title of an elaborate and interesting paper on a somewhat novel subject, presented by Dr. J. B. Murphy, of Chicago, at the Richmond meeting of the American Association of Obstetricians and Gynecologists, September, 1896. The paper is elaborately and handsomely illustrated and was issued in separate form as a reprint from the *Medical Record* of January 16, 1897.

---

PHYSICIANS' Blue Book is the title of a brochure of seventy-eight pages, containing a list of delinquent debtors made up from lists furnished by the physicians of Buffalo. It is issued by the Press Publishing Co., G. E. Livingston, manager, 604 D. S. Morgan building, Buffalo, N. Y. Every local physician should possess one of these invaluable lists.

---

THE *Revista de Ciencias Medicas de Barcelona* begins its twenty-third year by donning a new dress of the latest styles of the typographer's art. It may now be classed as one of the neatest and cleanest of foreign journals and without doubt the ablest medical journal of Spain.

---

THE Illinois State Reformatory has lately issued its third biennial report. It is a handsomely printed and well-bound book of 118 pages, done at the reformatory—printing trade—school, Pontiac, Ill. We are indebted to Col. Chas. E. Felton, formerly of Buffalo, now one of the board of managers, for a copy of the report, which is an excellent record of valuable work done in the direction of reforming criminals.

The Buffalo General Hospital has recently issued its thirty-eighth annual report, covering the year 1896. It is a well-printed brochure of eighty-seven pages, from the press of Baker, Jones & Co., Buffalo, and contains interesting financial and business statements ; report of the training-school for nurses ; annual report of the secretary of the ladies' hospital association ; tables of medical and surgical diseases treated during the year ; and a list of physicians who have served as internes since the hospital was organised in 1858. It betrays a little careless proof-reading, as instanced on page 71, where "ciliotomies" and "ciliotomy" appear as near neighbors, and on page 88, O. J. Steele should read A. J. Steele. Dr. Steele's residence is St. Louis instead of Rochester. Dr. Newton L. Bates is a medical director of the U. S. navy, and his address is Washington, D. C.

---

We noted some time ago the refusal of William Wood & Co. to include the American Medical Publishers' Association in its list of medical societies, and it now gives us pleasure to note that D. Appleton & Co. do include it among the society meetings of interest to both the doctor and the advertiser—*Bulletin American Medical Publishers' Association.*

If we are to understand by the foregoing that Messrs. Wood & Co. decline to publish in their standard record of state and national societies in America the notice referred to, we think they were quite right in so doing ; if, on the other hand, they refuse to publish a notice of the meeting in their news columns the *Bulletin* has just cause for criticism. It is a mistake in our view to interweave the professional and business interests of medical journalism too closely.

